# Spin–torque generator engineered by natural oxidation of Cu

**DOI:** 10.1038/ncomms13069

**Published:** 2016-10-11

**Authors:** Hongyu An, Yuito Kageyama, Yusuke Kanno, Nagisa Enishi, Kazuya Ando

**Affiliations:** 1 Department of Applied Physics and Physico-Informatics, Keio University, 3-14-1 Hiyoshi, Yokohama 223-8522, Japan; 2 PRESTO, Japan Science and Technology Agency, Kawaguchi, Saitama 332-0012, Japan

## Abstract

The spin Hall effect is a spin–orbit coupling phenomenon, which enables electric generation and detection of spin currents. This relativistic effect provides a way for realizing efficient spintronic devices based on electric manipulation of magnetization through spin torque. However, it has been believed that heavy metals are indispensable for the spin–torque generation. Here we show that the spin Hall effect in Cu, a light metal with weak spin–orbit coupling, is significantly enhanced through natural oxidation. We demonstrate that the spin–torque generation efficiency of a Cu/Ni_81_Fe_19_ bilayer is enhanced by over two orders of magnitude by tuning the surface oxidation, reaching the efficiency of Pt/ferromagnetic metal bilayers. This finding illustrates a crucial role of oxidation in the spin Hall effect, opening a route for engineering the spin–torque generator by oxygen control and manipulating magnetization without using heavy metals.

## Abstract (web summary)

In thin film spintronic devices, heavy metals with strong spin-orbit coupling are required to achieve a sizeable spin Hall effect. Here, the authors demonstrate an enhancement of the spin Hall effect in Cu, a material with weak spin-orbit coupling, via natural oxidation.

## Graphical abstract

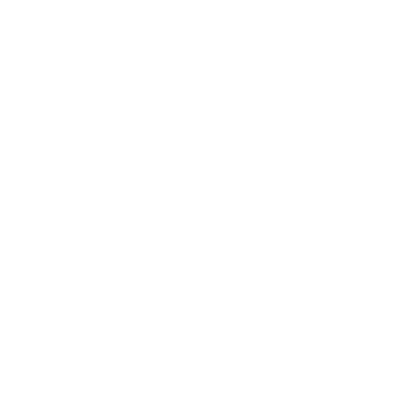

Interconversion between spin and charge currents is an essential building block of spin-based technologies^1^,^2^. A promising route for the interconversion is the direct use of spin–orbit interaction, which couples the spin of an electron to its momentum^3^,^4^,^5^,^6^,^7^,^8^,^9^,^10^,^11^. In the presence of the spin–orbit interaction, a non-zero spin current is generated in a direction perpendicular to an applied charge current, which is known as the direct spin Hall effect (DSHE)^12^,^13^,^14^,^15^,^16^,^17^,^18^,^19^. The spin–orbit interaction also causes the inverse process of the DSHE, a process that converts a spin current into a charge current: the inverse SHE^20^,^21^,^22^,^23^,^24^,^25^,^26^,^27^,^28^,^29^,^30^,^31^,^32^. The SHEs enable electric generation and detection of spin currents, offering new concepts of spintronic devices: spin Hall devices^33^, such as SHE transistors^34^, spin photodetectors^35^,^36^, spin thermoelectric converters^37^,^38^ and spin Hall magnetic memories^18^.

A key challenge for the development of such spin Hall devices is to achieve efficient conversion between spin and charge currents. However, to realize efficient spin-charge conversion, it has been believed that heavy metals with strong spin–orbit interaction are indispensable. This largely limits the selection of materials for the practical application of the spin Hall devices. Typically, a noble metal, Pt with around 10% conversion efficiency between spin and charge currents, has been used in most previous studies as a detector of spin currents or a generator of spin torque for magnetization manipulation. On the other hand, light metals have been confirmed to exhibit negligible SHEs. For instance, the conversion efficiency of Cu, a representative light metal with weak spin–orbit coupling, has been quantified to be two orders of magnitude smaller than that of Pt^9^,^39^, which has precluded using this low-cost light metal as a spin-charge converter. Thus, whether the SHEs can be enhanced in light metals is an important fundamental and practical question to push forward the application of the spin Hall devices with a large selection of materials.

In this study, we demonstrate that Cu becomes an efficient spin–torque generator through natural oxidation. This is evidenced by measuring spin–torque ferromagnetic resonance (ST-FMR) for Cu/Ni_81_Fe_19_ bilayers. The ST-FMR results show that the spin–torque generation efficiency from the Cu layer can be tuned by controlling the surface oxidization. We found that the maximum spin–torque generation efficiency in the naturally oxidized Cu/Ni_81_Fe_19_ bilayer is comparable to that in Pt/ferromagnetic metal bilayers. Our results also indicate that the observed spin–orbit torque in the naturally oxidized Cu/Ni_81_Fe_19_ bilayer cannot be attributed to interfacial spin–orbit coupling or the Rashba spin splitting. Thus, the efficient spin–torque generation revealed in the Cu/Ni_81_Fe_19_ bilayer demonstrates significant enhancement of the DSHE through the natural oxidation of Cu. These results provide a way for engineering the spin–torque generator driven by the DSHE through oxidation control.

## Results

### Spin–torque FMR

**Figure 1:**
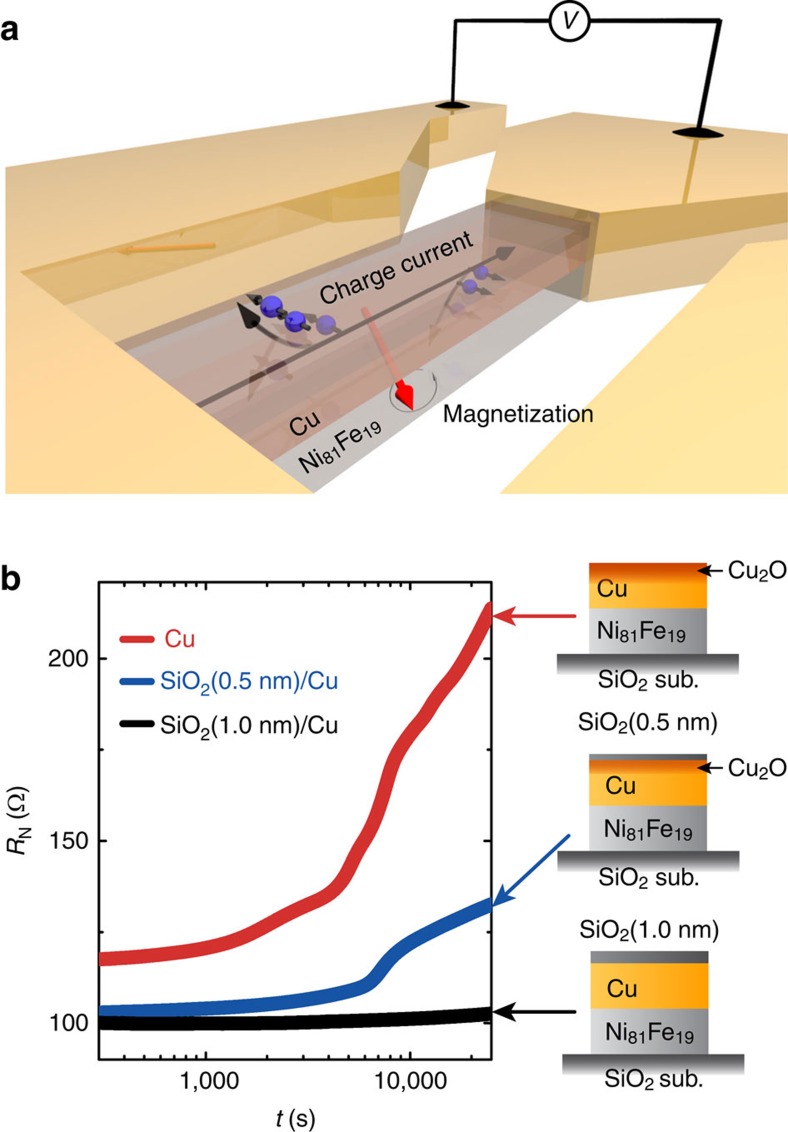
Device structure. (**a**) Schematic of a Cu/Ni_81_Fe_19_ bilayer for the ST-FMR measurements. The red arrow represents precessing magnetization in the Ni_81_Fe_19_ layer. The black arrows indicate the flow of conduction electrons in the Cu layer. An RF charge current was applied along the longitudinal direction of the Cu/Ni_81_Fe_19_ bilayer. (**b**) The time evolution of the electrical resistance *R*_N_ of the Cu layers capped with different thickness SiO_2_. The Cu layer was fabricated on a 8 nm-thick Ni_81_ Fe_19_ film and *R*_N_ of the Cu layer was extracted from the electrical resistance of the SiO_2_/Cu/Ni_81_ Fe_19_ film. The sample size for the resistance measurement was designed as same as that used in the ST-FMR measurements. The time *t*=0 was defined as the moment when the lift-off procedure was finished for each sample.

We use the ST-FMR technique to determine the generation efficiency of the spin–orbit torques affected by the natural oxidation of Cu/Ni_81_Fe_19_ bilalyers^13^. In the ST-FMR experiment, a microwave-frequency charge current is applied along the longitudinal direction of the device and an in-plane external magnetic field *μ*_0_*H* is applied with an angle of 45° from the longitudinal direction of the device as shown in Fig. 1a. The radio frequency (RF) current in the Cu layer generates an oscillating transverse spin current through the DSHE and then is injected into the adjacent Ni_81_Fe_19_ layer. The magnetization of the Ni_81_Fe_19_ layer is influenced by two torques generated from the RF charge current, an in-plane torque and an out-of-plane torque^13^. When the microwave frequency and the external magnetic field satisfy the FMR condition, the magnetization precession driven by the two torques will result in an oscillation of the resistance due to the anisotropic magnetoresistance in the Ni_81_Fe_19_ layer. By using a bias tee, a DC voltage signal across the device from the mixing of the RF current and oscillating resistance can be measured simultaneously during the microwave current application.

The Cu/Ni_81_Fe_19_ bilayer films used in the ST-FMR measurement were deposited by magnetron sputtering (for details, see Methods). Photolithography and lift-off techniques were used to pattern the films into rectangular strips with 10 μm width and 150 μm length. To tune the natural oxidation level in the Cu layers, the surfaces of the Cu layers were capped by SiO_2_ films with different thicknesses using sputtering from a compositional target. This method to control the natural oxidation has been well studied previously^40^. In fact, in the Cu/Ni_81_Fe_19_ bilayers, the time evolution of the electrical resistance *R*_N_ of the Cu layer due to the natural oxidation depends on the thickness of the SiO_2_ capping layer as shown in Fig. 1b, indicating that the oxidation level in the Cu layer can be tuned by the thickness of the capping layer as well as the time the bilayer exposed to the laboratory ambient.

**Figure 2:**
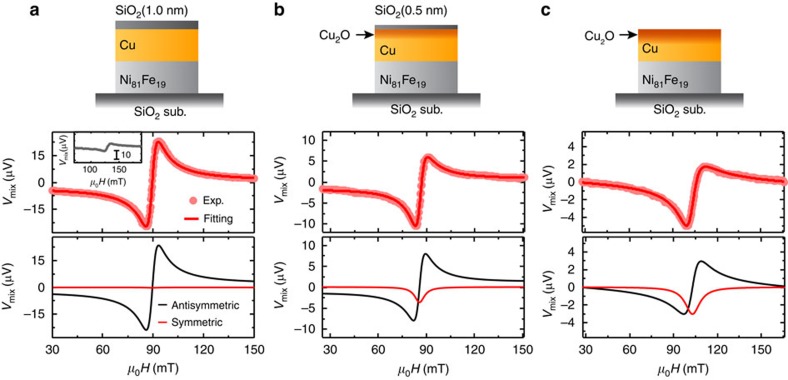
ST-FMR spectra of the Cu/Ni_81_Fe_19_ bilayers. The magnetic field *μ*_0_*H* dependence of the DC voltage *V*_mix_ at 7 GHz for the (**a**) SiO_2_(1 nm)/Cu(10 nm)/Ni_81_Fe_19_(8 nm), (**b**) SiO_2_(0.5 nm)/Cu(10 nm)/Ni_81_Fe_19_(8 nm) and (**c**) Cu(10 nm)/Ni_81_Fe_19_(8 nm) devices. The schematic illustrations of the natural oxidation manipulation in the Cu layers by changing the thickness of the SiO_2_ capping layer are also shown. The RF power of 24.7 dBm was applied for all the measurements. The solid circles are the experimental data and the solid curves are the fitting result using equation (1). The inset in **a** shows the *V*_mix_ spectrum for the SiO_2_(4 nm)/Ni_81_Fe_19_(8 nm) film at 7 GHz. The symmetric and antisymmetric components of the fitting results are plotted correspondingly.

Figure 2a shows the ST-FMR spectra of the Cu(10 nm)/Ni_81_Fe_19_(8 nm) bilayer capped with a 1.0 nm-thick SiO_2_ film measured at a frequency of 7.0 GHz. As shown in Fig. 2a, the DC voltage signal *V*_mix_ significantly changes under the FMR condition. In the ST-FMR measurement, *V*_mix_ is expressed as^13^,^41^


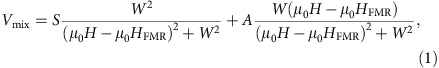


where *S*, *A*, *W* and *μ*_0_*H*_FMR_ are the magnitude of the symmetric component, the magnitude of the antisymmetric component, the spectral width and the FMR field, respectively. As shown in Fig. 2a, the experimental results are well reproduced using equation (1) (see the red curve). Based on the fitting result, the symmetric and antisymmetric voltage signals are also plotted separately in the lower part of Fig. 2a. Here, the symmetric part is proportional to the damping-like spin–orbit torque and the antisymmetric part is due to the sum of the Oersted field torque and the field-like spin–orbit torque^42^. Thus, the negligible symmetric voltage shown in Fig. 2a indicates inefficient generation of the damping-like torque in the SiO_2_(1 nm)/Cu(10 nm)/Ni_81_Fe_19_(8 nm) device, consistent with the weak DSHE in Cu, which is protected from surface oxidation.

To directly compare the influence of the oxidation level on the spin–torque generation, we measured the ST-FMR for the Cu/Ni_81_Fe_19_ films with different thicknesses of the SiO_2_ capping layer. Notably, as shown in Fig. 2b, the symmetric component of the voltage signal appears by reducing the thickness of the SiO_2_ capping layer from 1.0 to 0.5 nm. Furthermore, the ratio of the symmetric voltage to the antisymmetric voltage further increases in the Cu/Ni_81_Fe_19_ film, where the capping SiO_2_ layer is absent, as shown in Fig. 2c. These results demonstrate that the spin–torque generation efficiency is enhanced by the natural oxidation in the Cu/Ni_81_Fe_19_ bilayer, because the oxidation level of the Cu layer is tuned by the thickness of the SiO_2_ layer. We also studied the voltage generation for a single-layer Ni_81_Fe_19_(8 nm) film capped with a 4 nm-thick SiO_2_ as shown in the inset of Fig. 2a. The Ni_81_Fe_19_ film gives a purely antisymmetric signal, which arises from an Oersted field induced by the non-uniform current flow at the ends of the Ni_81_Fe_19_ due to the electrode contacts^13^,^43^. The lack of field-symmetric components in the resonance curve supports that the symmetric voltage signals observed in the naturally oxidized Cu/Ni_81_Fe_19_ bilayers arise from the oxidized Cu layer.

**Figure 3:**
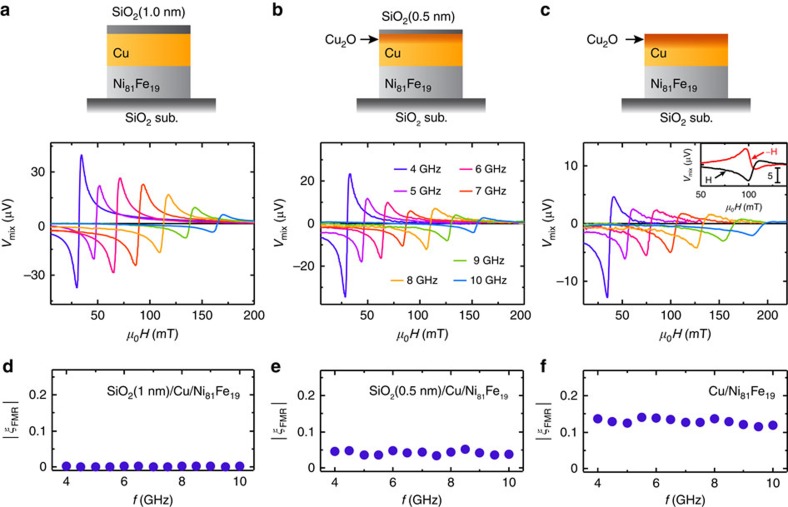
Spin–torque generation efficiency. The ST-FMR spectra for the (**a**) SiO_2_(1 nm)/Cu(10 nm)/Ni_81_Fe_19_(8 nm), (**b**) SiO_2_(0.5 nm)/Cu(10 nm)/Ni_81_Fe_19_(8 nm) and (**c**) Cu(10 nm)/Ni_81_Fe_19_(8 nm) devices for different RF current frequencies from 4 to 10 GHz. The inset in **c** shows the ST-FMR spectra at 7 GHz for both positive *H* and negative *H* magnetic fields. The FMR spin–torque efficiency *ξ*_FMR_ for the (**d**) SiO_2_(1 nm)/Cu(10 nm)/Ni_81_Fe_19_(8 nm), (**e**) SiO_2_(0.5 nm)/Cu(10 nm)/Ni_81_Fe_19_(8 nm) and (**f**) Cu(10 nm)/Ni_81_Fe_19_(8 nm) devices calculated using equation (2).

To quantitatively investigate the influence of the oxidation in the Cu layer on the spin–torque generation efficiency, we have systematically measured the ST-FMR spectra by changing the current frequency from 4 to 10 GHz as shown in Fig. 3a–c. We have confirmed that the symmetric component of the observed voltage changes the sign by reversing the magnetic field direction (see the inset to Fig. 3c). This sign reversal is consistent with the prediction of the spin–torque-driven FMR^13^. By fitting the measured voltage using equation (1) and using^13^,^41^


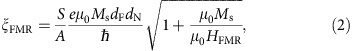


we determined the FMR spin–torque efficiency *ξ*_FMR_ for the Cu/Ni_81_Fe_19_ bilayers as shown in Fig. 3d–f, where *d*_F_ and *d*_N_ are the thicknesses of the Ni_81_Fe_19_ layer and the Cu layer, respectively. Here, the saturation magnetization *M*_s_ was obtained from the microwave frequency *f* dependence of the FMR field *μ*_0_*H*_FMR_ using the Kittel formula: 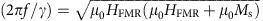, where *γ* is the gyromagnetic ratio.

Figure 3 demonstrates significant enhancement of *ξ*_FMR_, or the spin–torque generation efficiency, by the natural oxidation of the Cu layers. By averaging all the measured values of *ξ*_FMR_ at different frequencies, we obtain *ξ*_FMR_=0.13±0.0079 for the Cu/N_81_Fe_19_ bilayer without the SiO_2_ capping layer (see Fig. 3f). This large value is more than two orders of magnitude larger than that for the SiO_2_(1 nm)/Cu/N_81_Fe_19_ device (*ξ*_FMR_=0.00087±0.00032), where the Cu layer is protected from the natural oxidation (see Fig. 3a,d). The experimentally measured FMR spin–torque efficiency *ξ*_FMR_ is related to the damping-like *ξ*_DL_ and field-like *ξ*_FL_ spin–torque efficiencies as^42^


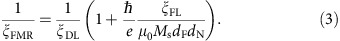


**Figure 4:**
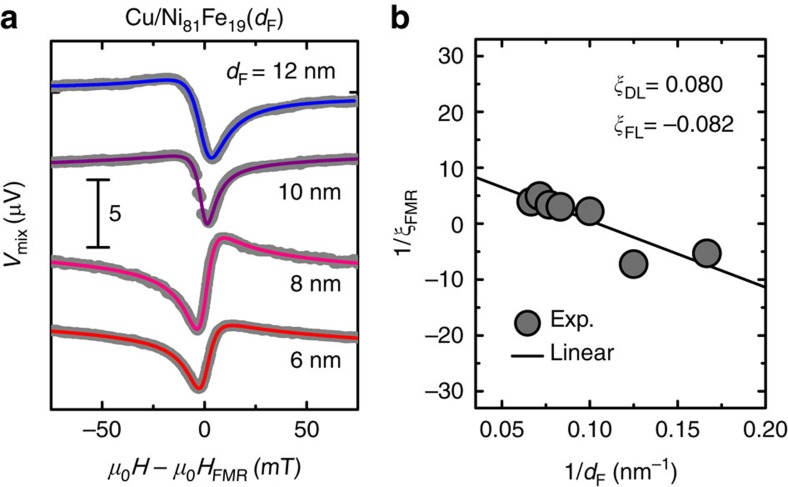
Thickness dependence of ST-FMR. (**a**) The ST-FMR spectra for the naturally oxidized Cu(10 nm)/Ni_81_Fe_19_(*d*_F_) bilayers at 7 GHz. *d*_F_ is the thickness of the Ni_81_Fe_19_ layer. (**b**) The inverse of the FMR spin–torque efficiency 1/*ξ*_FMR_ as a function of 1/*d*_F_ for the naturally oxidized Cu/Ni_81_Fe_19_ bilayer. The solid circles are the experimental data and the solid line is the linear fit to the data.

To determine *ξ*_DL_ and *ξ*_FL_, we measured Ni_81_Fe_19_ layer thickness *d*_F_ dependence of the ST-FMR spectra for the naturally oxidized Cu/Ni_81_Fe_19_ bilayer (see Fig. 4a,b). Figure 4a shows that the sign of the antisymmetric component of *V*_mix_ changes by changing the thickness of the Ni_81_Fe_19_ layer, indicating the presence of both the field-like torque and the Oersted torque with opposite signs^42^. From the 1/*d*_F_ dependence of 1/*ξ*_FMR_ shown in Fig. 4b with equation (3), we obtain *ξ*_DL_=0.080 and *ξ*_FL_=−0.082 for the naturally oxidized Cu/Ni_81_Fe_19_ bilayer. The positive sign for *ξ*_DL_ corresponds to the same sign of the damping-like torque as for Pt and the minus sign for *ξ*_FL_ indicates that the field-like effective field is opposite to the Oersted field^44^. The generation efficiency of the damping-like torque *ξ*_DL_=0.080 of the naturally oxidized Cu/Ni_81_Fe_19_ bilayer is comparable to that of Pt/ferromagnetic metal bilayers (see also Supplementary Fig. 1 and Supplementary Note 1)^42^.

## Discussion

Our experimental finding is that the spin–torque generation efficiency of the Cu/Ni_81_Fe_19_ bilayer is significantly enhanced by the natural oxidation; the spin–torque generation efficiency of the naturally oxidized Cu/Ni_81_Fe_19_ bilayer is comparable to that of Pt/ferromagnetic metal bilayers. There are two possible scenarios of the efficient spin–torque generation from the naturally oxidized Cu: the spin–torque generation by the bulk SHE or the interfacial Rashba effect.

**Figure 5:**
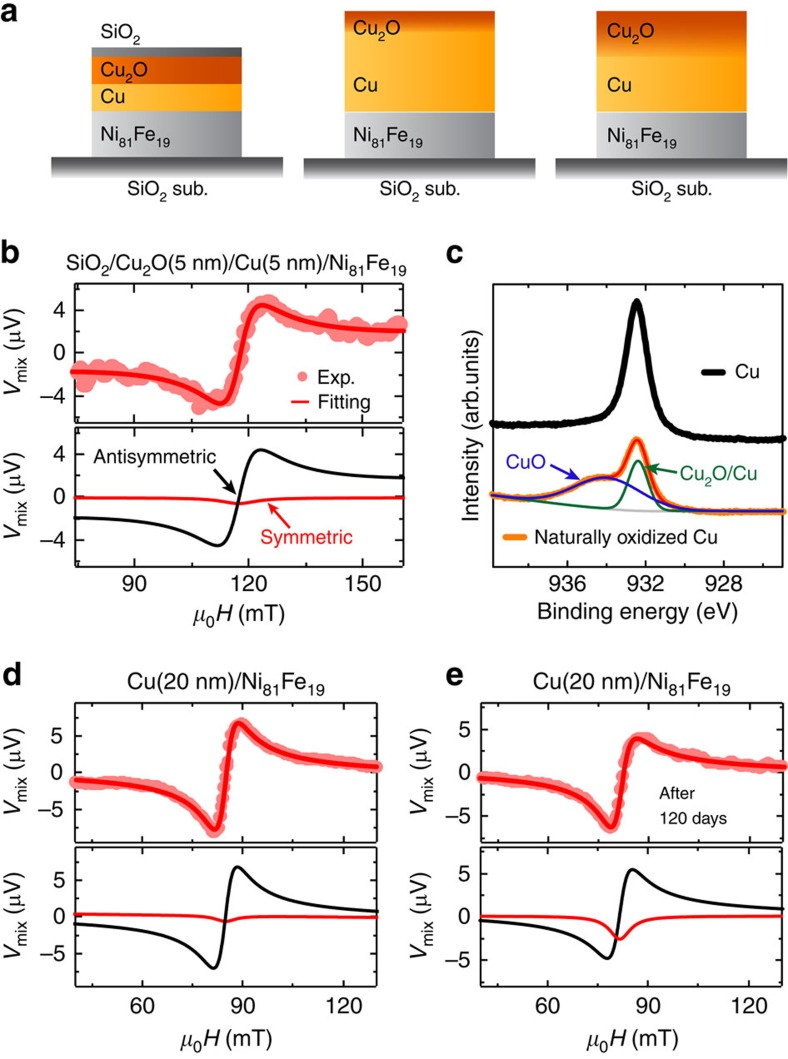
ST-FMR in different systems and oxidation characterization. (**a**) Schematic illustrations for the SiO_2_/Cu_2_O(5 nm)/Cu(5 nm)/Ni_81_Fe_19_, Cu(20 nm)/Ni_81_Fe_19_ and Cu(20 nm)/Ni_81_Fe_19_(120 days after the fabrication) devices, respectively. (**b**) The measured and fitted ST-FMR spectra of the SiO_2_/Cu_2_O(5 nm)/Cu(5 nm)/Ni_81_Fe_19_(8 nm) film. (**c**) Comparison of the X-ray photoelectron spectra for Cu and naturally oxidized Cu films with thickness of 10 nm. (**d**) The measured and fitted ST-FMR spectra of the Cu(20 nm)/Ni_81_Fe_19_(8 nm) film at 7 GHz. (**e**) The measured and fitted ST-FMR spectra of the Cu(20 nm)/Ni_81_Fe_19_(8 nm) film at 7 GHz, where the device was exposed to the laboratory ambient for 120 days after the fabrication.

The Rashba effect is unlikely to be the origin of the spin–torque observed in the Cu/Ni_81_Fe_19_ bilayer. As the Rashba spin splitting arises from electronic discontinuities at interfaces between two distinct materials, the Rashba effect in the naturally oxidized Cu/Ni_81_Fe_19_ bilayer can be induced at two interfaces: the Cu/Ni_81_Fe_19_ and oxidized Cu/Cu interfaces. First, the spin–torque due to the Rashba effect at the Cu/Ni_81_Fe_19_ interface is negligible in the naturally oxidized Cu/Ni_81_Fe_19_ bilayer. In the naturally oxidized Cu/Ni_81_Fe_19_ bilayer, the Cu/Ni_81_Fe_19_ interface is not affected by the natural oxidation, as the surface oxidation in the Cu/Ni_81_Fe_19_ bilayer used for the ST-FMR measurement can be as much as 4 nm into the bulk and the interface is barely affected by the oxidation (see also Methods)^45^. Therefore, the Rashba spin–torque generated at the Cu/Ni_81_Fe_19_ interface in the naturally oxidized Cu/Ni_81_Fe_19_ bilayer can be estimated from the ST-FMR result for the SiO_2_-covered Cu/Ni_81_Fe_19_ bilayer. As shown in Fig. 3d, the spin–torque in the SiO_2_(1 nm)/Cu/Ni_81_Fe_19_ device is negligible, demonstrating negligible Rashba spin torque at the Cu/Ni_81_Fe_19_ interface in the naturally oxidized Cu/Ni_81_Fe_19_ bilayer. Second, the Rashba effect that can be induced at the oxidized Cu/Cu interface also does not produce sizable spin–torque enough to explain the experimental observation. The Rashba effect, characterized by the Rashba coefficient *α*_R_, creates a three-dimensional spin current 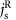 from an interfacial 2D charge current 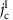 as ref. 46


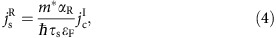


where *τ*_s_ is the effective relaxation time, m* is the effective electron mass and *ɛ*_F_ is the Fermi energy. In the presence of the Rashba spin splitting at the oxidized Cu/Cu interface, the spin current 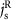 generated at the oxidized Cu/Cu interface diffuses through the Cu layer, which can give rise to a spin–torque on the magnetization in the Ni_81_Fe_19_ layer. To estimate the lowest value of the Rashba coefficient *α*_R_ required to reproduce the experimental result 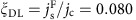, we neglect the spin relaxation and spin memory loss in the naturally oxidized Cu/Ni_81_Fe_19_ bilayer: 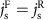, where 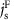 is the spin current density injected into the Ni_81_Fe_19_ layer and *j*_c_ is the charge current density in the naturally oxidized Cu layer. Using 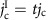, where *t*=0.5 nm is the interface thickness^47^,^48^, and equation (4) with m*=9.20 × 10^−31^ kg, *τ*_s_=0.5 × 10^−14^ s and *ɛ*_F_=7.0 eV^47^,^49^, we obtain the Rashba coefficient that reproduces the observed *ξ*_DL_=0.080 as *α*_R_=6.5 eVÅ. This value, the lower bound of *α*_R_, is much larger than the largest value of the Rashba coefficient observed in metallic systems so far^46^. Therefore, this result indicates that the Rashba effect at the oxidized Cu/Cu interface is irrelevant to the efficient spin–torque generation in the naturally oxidized Cu/Ni_81_Fe_19_ bilayer. Further evidence of the negligible spin–torque due to the Rashba effect at the oxidized Cu/Cu interface is obtained by measuring the ST-FMR for a Cu_2_O/Cu/Ni_81_Fe_19_ trilayer, where the surface of the Cu layer is capped by Cu_2_O. The Cu_2_O layer was deposited by applying oxygen with a 3% flow ratio into argon gas during the sputtering (for details, see Methods). After the Cu_2_O deposition, a SiO_2_ layer was capped on the film to prevent further oxidation (see Fig. 5a). Although both Cu_2_O/Cu/Ni_81_Fe_19_ trilayer and naturally oxidized Cu/Ni_81_Fe_19_ bilayer have the oxidized Cu/Cu interface, we found that the spin–torque generation efficiency in the Cu_2_O/Cu/Ni_81_Fe_19_ trilayer is more than an order of magnitude smaller than that in the naturally oxidized Cu/Ni_81_Fe_19_ bilayer as shown in Fig. 5b. This result supports that the diffusive spin current generated by the Rashba effect at the oxidized Cu/Cu interface is not responsible for the efficient spin–torque generation in the naturally oxidized Cu/Ni_81_Fe_19_ bilayer. This result also demonstrates a negligible non-local Rashba–Edelstein field, where the spin accumulation generated at the Rashba interface exchange couples to the magnetization in the Ni_81_Fe_19_ layer^50^. We also note that in the naturally oxidized Cu/Ni_81_Fe_19_ bilayer, electrons in the Cu layer reflected from the oxidized Cu/Cu interface can become spin-polarized due to the spin–orbit scattering and then enter the Ni_81_Fe_19_ layer with their spin being non-collinear to the magnetization^51^. This process can also generate the damping-like torque. However, the negligible symmetric voltage in the ST-FMR spectrum for the Cu_2_O/Cu/Ni_81_Fe_19_ trilayer indicates that the spin torque due to the spin–orbit scattering at the oxidized Cu/Cu interface is negligible. Thus, the efficient spin–torque generation in the naturally oxidized Cu/Ni_81_Fe_19_ bilayer cannot be attributed to the spin–orbit scattering, as well as the Rashba effect at the oxidized Cu/Cu interface.

The above result shows that the efficient spin–torque generation in the naturally oxidized Cu/Ni_81_Fe_19_ bilayer arises from significant enhancement of the DSHE in the Cu layer through the natural oxidation. Although the exact mechanism responsible for the enhancement is not understood in details, the dominant contribution to the DSHE in the naturally oxidized Cu layer can be attributed to spin-dependent scatterings in the interior of the Cu layer rather than the surface scatterings. The surface scattering strongly affects the spin-dependent transport in Cu because of the long mean free path of the conduction electrons. In fact, the spin relaxation length of Cu is known to be strongly dependent on the thickness^52^. For the SHEs, an important role of the surface scattering has been reported for Au^53^. However, the critical difference in the spin–torque generation efficiency between the Cu_2_O/Cu/Ni_81_Fe_19_ trilayer and naturally oxidized Cu/Ni_81_Fe_19_ bilayer indicates that the difference in the surface scattering between the oxidized Cu/Cu and SiO_2_/Cu interfaces is not the main source of the enhancement of the DSHE induced by the surface oxidation.

In the naturally oxidized Cu, an applied charge current flows in a partially oxidized Cu layer and in the non-oxidized Cu layer. The natural oxidation process creates a duplex-type oxidized structure, CuO/Cu_2_O/Cu, of an insulating CuO layer at the surface and an inner semiconducting Cu_2_O layer^54^. In fact, as shown in Fig. 5c, the CuO peak can be clearly observed after natural oxidation of a Cu film in the X-ray photoelectron spectra. It has been confirmed that the oxygen concentration gradually decreases from the CuO/Cu_2_O interface to the interior of the Cu layer^55^; thus, precisely, the Cu_2_O layer is a mixture of Cu_2_O and non-oxidized Cu. With the surface- or interface-dominated Rashba and SHEs ruled out as mechanisms behind the efficient spin–torque generation in the naturally oxidized Cu/Ni_81_Fe_19_ bilayer, the only possible mechanism that agrees with the observed damping-like spin–torque is the DSHE in the mixed region of the Cu_2_O and non-oxidized Cu layer. Although detailed calculations are necessary to address the mechanism of the giant DSHE in the mixed region, we found that the spin–torque generation efficiency depends on the thickness of the Cu layer; the symmetric component of *V*_mix_ is negligible in a thicker naturally oxidized Cu(20 nm)/Ni_81_Fe_19_ bilayer as shown in Fig. 5d. We also found that by further exposing the Cu(20 nm)/Ni_81_Fe_19_ bilayer to the laboratory ambient, the ratio of the symmetric voltage to the antisymmetric voltage increases as shown in Fig. 5e. For the ST-FMR measurement shown in Fig. 5e, the Cu(20 nm)/Ni_81_Fe_19_ bilayer was exposed to the laboratory ambient for 120 days after the fabrication, which results in the effective thickness of the oxidized layer of around 6 nm^56^. From this result, we obtained *ξ*_FMR_=0.053, which is clearly enhanced from *ξ*_FMR_=0.011 obtained from the result shown in Fig. 5d, supporting that the natural oxidation is responsible for the spin–torque generation in the Cu/Ni_81_Fe_19_ bilayers. These results are consistent with the DSHE in the intermediate Cu_2_O layer, as in the thicker Cu film only a small amount of the applied charge current flows in the highly resistive layer of the mixture of Cu_2_O and non-oxidized Cu, and most of the charge current flows in the non-oxidized Cu layer with the negligible DSHE.

**Figure 6:**
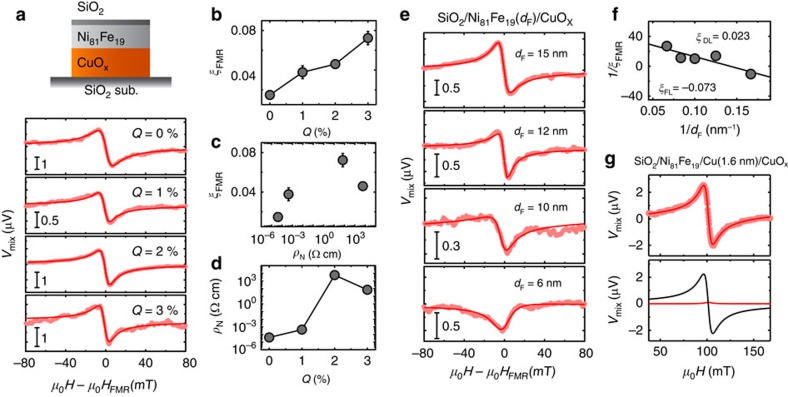
ST-FMR spectra of the Ni_81_Fe_19_/CuO_*x*_ bilayers. (**a**) The ST-FMR spectra for the SiO_2_(4 nm)/Ni_81_Fe_19_(8 nm)/CuO_*x*_(15.9 nm) films with different *Q* at 7 GHz. (**b**) The *Q* dependence of *ξ*_FMR_ for the SiO_2_/Ni_81_Fe_19_/CuO_*x*_. The error bars are defined as s.e.m. (**c**) *ξ*_FMR_ for the SiO_2_/Ni_81_Fe_19_/CuO_*x*_ device as a function of the electrical resistivity *ρ*_N_ of the CuO_*x*_ layer. (**d**) The *Q* dependence of the electrical resistivity *ρ*_N_ of the CuO_*x*_ layer. (**e**) The ST-FMR spectra for the SiO_2_/Ni_81_Fe_19_/CuO_*x*_ with *Q*=3% for different *d*_F_ at 7 GHz, where *d*_F_ is the thickness of the Ni_81_Fe_19_ layer. (**f**) The inverse of the FMR spin–torque efficiency 1/*ξ*_FMR_ as a function of 1/*d*_F_ for the SiO_2_/Ni_81_Fe_19_/CuO_*x*_. The solid circles are the experimental data and the solid line is the linear fit to the data. (**g**) The measured and fitted ST-FMR spectra of the SiO_2_(4 nm)/Ni_81_Fe_19_(8 nm)/Cu(1.6 nm)/CuO_*x*_(14.3 nm) film at 7 GHz.

To further clarify the role of the natural oxidation in the spin–torque generation, we measured the ST-FMR spectra for Ni_81_Fe_19_(8 nm)/CuO_*x*_(15.9 nm) bilayers, where the Cu layer was homogeneously oxidized using the reactive RF magnetron sputtering with the mixture of argon and oxygen gas. For the sputtering, the amount of oxygen gas flow *Q* in the reactive mixture was varied between 0 and 3%. To avoid the oxidation of the Ni_81_Fe_19_ layer, the CuO_*x*_ layer was first sputtered on a SiO_2_ substrate and then the Ni_81_Fe_19_ layer was sputtered on the CuO_*x*_ layer. The surface of the Ni_81_Fe_19_ layer was capped with a 4 nm-thick SiO_2_ film. Figure 6a shows the ST-FMR spectra for the SiO_2_/Ni_81_Fe_19_/CuO_*x*_ devices fabricated with different amount of the oxygen gas flow *Q*. Using this result, we plotted the FMR spin–torque efficiency *ξ*_FMR_ for the SiO_2_/Ni_81_Fe_19_/CuO_*x*_ as a function of *Q* in Fig. 6b. This result indicates that a damping-like torque appears in the SiO_2_/Ni_81_Fe_19_/CuO_*x*_ with increasing the homogeneous oxidation level of the Cu layer. Therefore, the spin–orbit torque can be generated not only from the inhomogeneously-oxidized Cu (naturally oxidized Cu) but also from the homogeneously oxidized Cu.

In Fig. 6c, we show *ξ*_FMR_ as a function of electrical resistivity *ρ*_N_ of the CuO_*x*_ layer. This result indicates that the FMR spin–torque efficiency does not scale with the electrical resistivity. As shown in Fig. 6d, the electrical resistivity of the Cu layer increases with increasing the oxygen flow *Q* during the sputtering due to the formation of Cu_2_O. Figure 6d also shows that the further increase of *Q* results in the lowering of the electrical resistivity. The lowering of the resistivity has been explained as resulting from the excess oxygen doping, which effectively produces more copper ion vacancies in the *p*-type semiconductor Cu_2_O (ref. 57). Thus, these results demonstrate that the FMR spin–torque efficiency in the SiO_2_/Ni_81_Fe_19_/CuO_*x*_ is dominated by the oxidation level of the Cu layer rather than the electrical resistivity of the CuO_*x*_ layer.

In contrast to the spin–torque generation in the naturally oxidized Cu/Ni_81_Fe_19_ bilayer, the origin of the spin–torque observed in the SiO_2_/Ni_81_Fe_19_/CuO_*x*_ bilayer cannot be attributed to the DSHE in the homogeneously oxidized Cu layer. Here, it is noteworthy that at *Q*=3% the electrical resistance of the CuO_*x*_ layer exceeds 3 × 10^8^ Ω, which is six orders of magnitude larger than that of the Ni_81_Fe_19_ layer. Thus, most of the applied charge current flows in the Ni_81_Fe_19_ layer and only a fraction of the charge current flows in the CuO_*x*_ layer. The negligible charge current in the CuO_*x*_ layer cannot generate the sizable symmetric voltage or the damping-like torque, through the DSHE in the SiO_2_/Ni_81_Fe_19_/CuO_*x*_ devices. To determine the damping-like *ξ*_DL_ and field-like *ξ*_FL_ spin–torque efficiencies in the SiO_2_/Ni_81_Fe_19_/CuO_*x*_ structure with *Q*=3%, we measured the ST-FMR spectra for different Ni_81_Fe_19_ layer thickness *d*_F_ as shown in Fig. 6e. In Fig. 6f, we show 1/*ξ*_FMR_ as a function of 1/*d*_F_. From this result with equation (3), we obtain *ξ*_DL_=0.023 and *ξ*_FL_=−0.073 for the SiO_2_/Ni_81_Fe_19_/CuO_*x*_ with *Q*=3%. The efficient generation of the field-like torque, rather than the damping-like torque, supports that the spin–torque arises not from the DSHE in the CuO_*x*_ layer but from the Rashba effect^48^.

The difference in the naturally oxidized Cu/Ni_81_Fe_19_ bilayer and the Ni_81_Fe_19_/CuO_*x*_ bilayer is the interface oxidation and the homogeneity of the oxidation level in the Cu layer. In the naturally oxidized Cu/Ni_81_Fe_19_ bilayer, the oxidation level of the Cu layer decreases exponentially from the surface and the Cu/Ni_81_Fe_19_ interface is not affected by the natural oxidation. In contrast, in the Ni_81_Fe_19_/CuO_*x*_ bilayer, the Ni_81_Fe_19_ layer is in direct contact with the oxidized Cu layer. This difference of the interface gives rise to the different mechanisms responsible for the spin–torque generation. In Fig. 6g, we show the ST-FMR spectrum for a SiO_2_/Ni_81_Fe_19_(8 nm)/Cu(1.6 nm)/CuO_*x*_(14.3 nm) device with *Q*=3%. This result demonstrates that the symmetric component of *V*_mix_, or the damping-like torque, becomes negligible by inserting the very thin Cu layer. From the result shown in Fig. 6g, the FMR spin–torque efficiency for the SiO_2_/Ni_81_Fe_19_/Cu(1.6 nm)/CuO_*x*_ is determined to be *ξ*_FMR_=0.009±0.002, which is about an order of magnitude smaller than that for the SiO_2_/Ni_81_Fe_19_/CuO_*x*_. This result supports that the direct contact between the Ni_81_Fe_19_ and CuO_*x*_ layers is essential for the spin–torque generation in the SiO_2_/Ni_81_Fe_19_/CuO_*x*_ device (see also Fig. 5b). This is clearly different from the spin–torque generation in the naturally oxidized Cu/Ni_81_Fe_19_ bilayer, as in the bilayer the sizable damping-like torque is observed, despite the fact that the Ni_81_Fe_19_ layer and oxidized Cu layer are separated by non-oxidized Cu. Although the DSHE in the interior of the CuO_*x*_ layer can be enhanced with the oxidation level as same as the naturally oxidized Cu, our results suggest that the spin–orbit torque generated by the Rashba effect at the Ni_81_Fe_19_/CuO_*x*_ interface exceeds that generated by the DSHE of the CuO_*x*_ layer in the Ni_81_Fe_19_/CuO_*x*_ bilayer. We also note that the ratio of the symmetric voltage to the antisymmetric voltage is almost independent of the thickness of the inserted Cu layer as shown in Figs 5b and 6g. In these devices, because of the high resistivity of the oxidized Cu layer, most of the applied current flows in the Cu layer. Thus, the effect of electron scatterings at the Cu/CuO_*x*_ and Ni_81_Fe_19_/Cu interfaces on the spin–torque generation is expected to become significant by decreasing the thickness of the inserted Cu layer. Nevertheless, we found negligible difference in the ST-FMR spectra for the trilayers with different Cu thicknesses. This demonstrates again that the spin–orbit scattering at the oxidized Cu/Cu and Ni_81_Fe_19_/Cu interfaces plays a minor role in the spin–torque generation in the naturally oxidized Cu/Ni_81_Fe_19_ bilayers.

The significant enhancement of the spin–torque demonstrates the crucial role of the natural oxidation in the DSHE. This finding is clearly different from recent observations of the interfacial Rashba spin–orbit torque affected by oxygen incorporation^40^,^58^. The damping-like spin–torque efficiency *ξ*_DL_ obtained from the ST-FMR measurements is related to the spin Hall angle *θ*_SHE_ as *ξ*_DL_=*j*_s_^F^/*j*_c_=*T*_int_*θ*_SHE_ when *d*_N_≫*λ*_N_, where *T*_int_ is the interfacial spin transparency and *λ*_N_ is the spin-diffusion length of the non-magnetic layer^59^. However, this simple model cannot be applied directly to the present naturally oxidized Cu/Ni_81_Fe_19_ bilayer, as the spin Hall angle, as well as the charge and spin transport properties in the naturally oxidized Cu film are spatially non-uniform. A detailed theoretical calculation, which takes into account the spatial inhomogeneity of the spin and charge transport, is necessary to provide a detailed physical mechanism responsible for the DSHE in Cu induced by natural oxidation. Therefore, the finding of the spin–torque generation in the naturally oxidized Cu/Ni_81_Fe_19_ bilayers will serve as a stimulation of in-depth theoretical studies of the spin transport and our results provide an important piece of information for deeper understanding of the spin–orbit physics in solids. Furthermore, the efficient spin–torque generator achieved by the natural oxidation provides a route for developing efficient spintronic devices through oxidation engineering, promising important advances in spintronics and oxide electronics.

## Methods

### Sample preparation

The films were deposited on thermally oxidized Si substrates by RF magnetron sputtering at room temperature. The base pressure in the chamber before the deposition was better than 1 × 10^−6^ Pa and the deposition pressure was 0.2 Pa. The Ni_81_Fe_19_, Cu and SiO_2_ films were deposited by applying argon gas with flow rate of 10 s.c.c.m. The Cu layer was sputtered from a 99.99% purity target. For the Cu_2_O deposition, oxygen and argon gases were applied with flow rate of 0.3 and 9.7 s.c.c.m., respectively. The film thicknesses were controlled by the deposition time with a pre-calibrated deposition rate. For the fabrication of the devices used in ST-FMR experiment, the substrates were patterned into 10 μm × 150 μm rectangular shape by standard photolithography before deposition and lift-off technique was used to take off the rest part of the films after deposition. The ST-FMR experiments were taken within 5 h for all the devices after the fabrication, except for the notified cases. For the comparison between Cu and naturally oxidized Cu films in the X-ray photoelectron spectroscopy, two Cu blanket films with the thickness of 10 nm were used, where one sample was measured within 1 h after the fabrication and the other one was exposed to the laboratory ambient for 5 days before the measurement. All measurements were conducted at room temperature.

### Electric resistance

The time evolution of the electrical resistance *R*_N_ of the Cu layer shown in Fig. 1b was measured using the conventional four-probe method for the Cu/Ni_81_Fe_19_ bilayers with different thickness SiO_2_. The resistance of the Ni_81_Fe_19_ layer was eliminated from the measured resistance of the bilayers. From the change of the resistance *R*_N_ of the Cu layer, the effective thickness of the oxidized layer can be estimated as ∼4 nm in the 10 nm-thick Cu layer at the time when the ST-FMR was measured. For the estimation, we neglected the gradual change of the oxidation level and the charge-current flow in the oxidized layer for simplicity. This result supports that the Cu/Ni_81_Fe_19_ interface is not affected by the natural oxidation of the Cu/Ni_81_Fe_19_ bilayers.

### Data availability

The data that support the findings of this study are available from the corresponding author on request.

## Additional information

**How to cite this article:** An, H. *et al*. Spin–torque generator engineered by natural oxidation of Cu. *Nat. Commun.* **7**, 13069 doi: 10.1038/ncomms13069 (2016).

## Supplementary Material

Supplementary Information Supplementary Figure 1, Supplementary Note 1 and Supplementary Reference
