# Supplementary material for: Spin–torque generator engineered by natural oxidation of Cu
Source: Nat Commun. 2016 Oct 11;7:13069. doi: 10.1038/ncomms13069 (PMC5062613; doi:10.1038/ncomms13069)
Supplement: Supplementary Information — Supplementary Figure 1, Supplementary Note 1 and Supplementary Reference [file ncomms13069-s1.pdf]

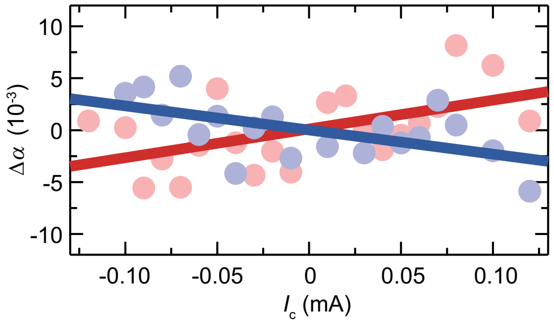

Supplementary Figure 1: Damping modulation. The change of the magnetization damping $\Delta\alpha$ for the naturally-oxidized Cu/$Ni_{81}Fe_{19}$ bilayer as a function of a DC charge current I_c. The red(blue) circles are the experimental data measured using ST-FMR with a positive(negative) external magnetic field. The solid lines are the linear fits to the data.

Supplementary Note 1: Damping modulation. We measured the magnetization damping α for the naturally-oxidized Cu/$Ni_{81}Fe_{19}$ bilayer as a function of a DC charge current. The dependence of the change of the damping constant $\Delta\alpha$ on the charge current density j_c in the naturally-oxidized Cu layer is expressed as[1]

$$\frac{\Delta\alpha}{j_\mathrm{c}} = \frac{\hbar}{2e}\frac{\xi_\mathrm{DL}}{\sqrt{2}M_\mathrm{s}d_\mathrm{F}(\mu_0 H_\mathrm{FMR} + 0.5\mu_0 M_\mathrm{s})}. \tag{1}$$

As shown in Supplementary Figure 1, from the linear fittings, the slopes $\Delta\alpha/j_c$ were obtained, and the damping-like torque efficiency ξ_{DL} was calculated as $\xi_{DL} = 0.03$, which is comparable to that obtained from the ST-FMR spectral shape. The difference in ξ_{DL} obtained from the ST-FMR spectral shape and damping modulation can be attributed to different spatial distribution of the RF and DC charge currents.

---

[1] L. Liu, T. Moriyama, D. Ralph, and R. Buhrman, Phys. Rev. Lett. **106**, 036601 (2011).
